# Vovel metrics—novel coupling metrics for improved software fault prediction

**DOI:** 10.7717/peerj-cs.590

**Published:** 2021-06-10

**Authors:** Rizwan Muhammad, Aamer Nadeem, Muddassar Azam Sindhu

**Affiliations:** 1 Department of Computer Science, Capital University of Science and Technology, Islamabad, Pakistan; 2 Department of Computer Science, Quaid-i-Azam University, Islamabad, Pakistan

**Keywords:** Expert opinion, Software coupling, Software faults, Software metrics

## Abstract

Software is a complex entity, and its development needs careful planning and a high amount of time and cost. To assess quality of program, software measures are very helpful. Amongst the existing measures, coupling is an important design measure, which computes the degree of interdependence among the entities of a software system. Higher coupling leads to cognitive complexity and thus a higher probability occurrence of faults. Well in time prediction of fault-prone modules assists in saving time and cost of testing. This paper aims to capture important aspects of coupling and then assess the effectiveness of these aspects in determining fault-prone entities in the software system. We propose two coupling metrics, i.e., Vovel-in and Vovel-out, that capture the level of coupling and the volume of information flow. We empirically evaluate the effectiveness of the Vovel metrics in determining the fault-prone classes using five projects, i.e., Eclipse JDT, Equinox framework, Apache Lucene, Mylyn, and Eclipse PDE UI. Model building is done using univariate logistic regression and later Spearman correlation coefficient is computed with the existing coupling metrics to assess the coverage of unique information. Finally, the least correlated metrics are used for building multivariate logistic regression with and without the use of Vovel metrics, to assess the effectiveness of Vovel metrics. The results show the proposed metrics significantly improve the predicting of fault prone classes. Moreover, the proposed metrics cover a significant amount of unique information which is not covered by the existing well-known coupling metrics, i.e., CBO, RFC, Fan-in, and Fan-out. This paper, empirically evaluates the impact of coupling metrics, and more specifically the importance of level and volume of coupling in software fault prediction. The results advocate the prudent addition of proposed metrics due to their unique information coverage and significant predictive ability.

## Introduction

Software is a complex entity, and its development needs careful planning and a large amount of time and cost. The software is human-dominated activity, therefore, errors are always there, and these errors can cause faults. In industrial projects, 15 to 50 faults per kilo lines of code (KLOC) are recorded, and in Microsoft’s applications, this figure is from 10 to 20 faults per KLOC (McConnell, 2004). Windows 2000 has about 63 thousand bugs in its 34 million line of code (MLOC) (ZDNet.net, 2000). Residual faults have a significant potential to cause a failure (Grice, 2015; Wakefield, 2016; Osborn, 2016). A consequence of failures can be from trivial inconvenience to catastrophic. Testing is a process that involves discovering the faults and thus preventing failure. However, exhaustive testing is required to reveal all the residual faults, due to which, testing exceeds 50% of the total development cost (Ammann & Offutt, 2008) and according to the IBM-reports, the cost can exceed 75% (Hailpern & Santhanam, 2002).

Generally, faults are not evenly distributed across the software product (Sherer, 1995). Some modules of a software product and are clustered in a limited number of modules (Sherer, 1995). Earlier studies show that sometimes faults are confined to only 42% of total software modules in a project (Gyimothy, Ferenc & Siket, 2005). A study by Ostrand, Weyuker & Bell (2004) on multiple releases of an inventory software system, reports that all faults (which could be found) are present in only 20% of total modules. According to Weyuker, Ostrand & Bell (2008), typically 20% of modules contain 80% of faults.

We expect that the testing process can significantly be assisted if the fault-prone (*fp*) modules are determined successfully. In this regard, the software fault prediction (SFP) plays a vital role by detecting *fp* module(s) or the number of expected faults in a software module. This is usually, accomplished by employing an artifact from the earlier release of the same software. The timely detection of faulty modules or the number of faults in any module is quite beneficial more especially, in critical and strategic software systems. It helps in reducing testing cost and improving the quality of the system. Moreover, this can direct the testing team to focus more on the fp modules. Predicting the number of faults can be even more useful as it provides the criteria for *sufficient testing*.

SFP works with metrics, which reflect some aspect of the software. Amongst these aspects, coupling is an important measure. As defined by Briand, Devanbu & Melo (1997), “Coupling refers to the degree of interdependence among the components of a software system”. A component can be a module of the system or a smaller entity such as a class or a method. Moreover, coupling can indicate a relation between two components but also a property of an entity compared to all the other related entities in the system. Over the years, different coupling measures have been proposed. Starting from structural metrics developed for procedural languages, new approaches were introduced to measure different relations in object-oriented environments. Nonetheless, the central importance of these metrics for software engineering encouraged researchers to propose even more coupling measures in an attempt to evaluate further connections between software entities. Therefore, research community is quite active in the derivation of new coupling metrics (Myers, 1975; Henry, 1979; Henry & Kafura, 1981; Chidamber & Kemerer, 1991; Offutt, Harrold & Kolte, 1993; Dhama, 1995; Binkley & Schach, 1997a; Harrison, Counsell & Nithi, 1998; Shao & Wang, 2003; Nachiappan & Thirumalesh, 2007; Miquirice & Wazlawick, 2018).

In principle, high coupling is undesirable, as increased coupling leads to increased complexity and consequent faults (Braude & Bernstein, 2016). The reason is that the highly coupled module is difficult to reuse, modify, or test without understanding all the modules to which it is coupled to. If an error occurs in a highly coupled module then the probability of an error in other modules increases. That is why highly coupled modules are more fault-prone (Tsui, Karam & Bernal, 2016). Another reason is that high coupling is difficult to comprehend by the developers. When coupling goes beyond the comprehensible level, programmer loses control and thus leads to the introduction of faults into the modules under development (Laplante, 2015). However, coupling due to inheritance promotes re-usability and it is not against modularization (Chidamber & Kemerer, 1991), and hence not considered in this study.

This article focuses on coupling’s impact on SFP. Such a direction can greatly help in integration testing. Test case prioritization may also be done by assigning a high priority to those test cases that cover strongly coupled modules. Likewise, test cases that cover the least coupled modules or isolated modules may be deferred for later execution. Numerous coupling metrics used in SFP (Zimmermann & Nagappan, 2008; Aggarwal et al., 2009; Kpodjedo et al., 2009; English et al., 2009; Jureczko & Spinellis, 2010; Malhotra, Kaur & Singh, 2010; Shatnawi, 2010; Elish, Al-Yafei & Al-Mulhem, 2011; Johari & Kaur, 2012; Rathore & Gupta, 2012; He et al., 2015; Kumari & Rajnish, 2015; Anwer et al., 2017), advocate for their predictive potential. However, the impact of coupling metrics with their key properties (like levels and volume) has not been evaluated yet

Keeping in view this, we designed two research questions which are shown in Table 1.

**Table 1:** Research questions and their objectives.

Q. No.	Research questions	Objective
RQ 1:	How much unique information is covered when coupling metric comprises volume and levels of coupling?	For analyzing the degree of unique information coverage when coupling is associated with volume and levels of coupling.
RQ 2:	What is the impact of volume and levels of coupling in SFP?	For analyzing the effectiveness of volume and levels of coupling in SFP

This paper focuses on evaluating the impact of volume and levels of coupling on the SFP. For this we propose two metrics, *Vovel-in* and *Vovel-out*, that incorporates the volume and levels of coupling. The Vovel metrics are assessed using five projects, i.e., Eclipse JDT, Equinox framework, Apache Lucene, Mylyn, and Eclipse PDE UI. Model building is done using univariate logistic regression and later Spearman correlation coefficient is performed with the existing coupling metrics to assess the coverage of unique information (RQ 1). Finally, the least correlated metrics are used for building multivariate logistic regression with and without the use of Vovel metrics, to assess the effectiveness of Vovel metrics, exclusively (RQ 2). Results concluded that our proposed coupling metrics significantly upgrade the performance to predict *fp* classes.

Rest of the paper is organized as follows: “Related Work” presents the literature review of this field. The proposed metrics have been discussed in “Vovel Metrics: Improved Coupling Metrics” followed by “Materials and Methods” that elaborates the materials and methods used to evaluate the proposed metric. Threats to validity of results are described in “Threats to Validity”. Finally, conclusion and future directions of the research are discussed in “Conclusion and Future Work”.

## Related Work

Research literature is quite rich in the derivation of new coupling metrics. These metrics have been used in various disciplines like in SFP, design patterns (Antoniol, Fiutem & Cristoforetti, 1998), re-modularization (Abreu, Pereira & Sousa, 2000), assessing software quality (Briand et al., 2000), maintenance cost (Akaikine, 2010), productivity (Sturtevant, 2013), software vulnerabilities (Lagerström et al., 2017), reusability (Hristov et al., 2012), changeability (Rongviriyapanish et al., 2016; Parashar & Chhabra, 2016; Kumar, Rath & Sureka, 2017), and reliability (Yadav & Khan, 2012).

In the context of SFP, the exclusive coupling has been addressed by many studies. Few of these studies are briefly discussed by Rizwan et al. in their recent studies (Rizwan, Nadeem & Sindhu, 2020a, 2020b). Kitchenham, Pickard & Linkman (1990) assessed multiple design metrics that are based on Henry and Kafura’s information flow metrics (i.e. Fan-in and Fan-out). A communication system was taken as a case study. The objective was to evaluate the ability of selected metrics to identify change-prone, error-prone, and complex programs. Based upon visual scatter plots, it was reported that the Fan-out has a strong association with software fault, whereas Fan-in is relatively weak in this trait.

Binkley & Schach (1998a, 1998b) investigated the usefulness of Coupling dependency metric (CDM), Ordinal scale module coupling(OSMC), Fan-in, and Fan-out in predicting run-time failures using Spearman correlation coefficient. OASIS was taken as a case study that is developed in COBOL. It was reported that the most accurate predictor of run-time failures is the amount of inter-dependency between modules, which is computed by the selected coupling metrics.

Briand et al. (1998) investigated the usefulness of existing coupling metrics in identifying the probability of fault detection. Both import and export couplings were used as independent variables. Medium-sized eight different software systems developed by students were used for the evaluation. Fault data was taken from the independent testing team. The experiment comprised CKs and Briand’s coupling metrics (Briand, Devanbu & Melo, 1997). The result of regression coefficients showed that all the coupling metrics are a good predictor of software faults except Briand’s OCMIC.

El Emam et al. (1999) examined the impact of CK and Briand’s coupling metrics in SFP after controlling the size of the software product. The dataset that was used came from the telecommunications software written in C++ having 85 classes. Metrics were parsed using static analysis tool and fault data was collected from the configuration management system. Model building was done using binary logistic regression. Results of *R*^2^ and coefficients showed that out of multiple coupling metrics in CK and Briand suite, only CBO, OCMEC, OCAEC, and OMMEC are good predictors of faults when controlling the size of the software product.

Tang, Kao & Chen (1999) evaluated CK metric suite using univariate logistic regression. They made three classes of faults; Object-oriented, Object management, and Traditional. They reported the usefulness of RFC. Moreover, the authors proposed few metrics and reported them useful as well.

Briand et al. (2000) explored the association of import/export coupling measures and the probability of fault detection. The eight systems used for this study were developed in C++ by students over the course of four months. The systems consist of 180 classes. Coupling metrics were parsed using M-System and fault data is collected during the testing phase which was conducted by an independent testing team. The authors concluded that coupling measures, with good variance, are significantly useful in predicting software faults. The result of univariate logistic regression showed that all import and export couplings are useful in SFP except OCAEC.

El Emam et al. (2001) applied logistic regression and Pearson correlation on the telecommunications framework written in C++. They evaluated the association of CBO, RFC, and Briand’s metric suite with software faults. They reported that CBO and RFC both, are associated with faults, whereas RFC’s association gets weaker when size is controlled.

Subramanyam & Krishnan (2003) investigated the performance of CBO (and some non-coupling metrics) in SFP. The study used an e-commerce application suite developed in C++ and Java, wherein the total classes are 706. Metrics were computed from the design document and source code. Fault data was collected from customer acceptance testing and fault resolution logs, which were later validated by the concerned development team. They examined the effect of the size along with the CBO values on the faults by employing multivariate regression. Besides validating the usefulness of metrics, they compared the applicability of the metrics in different languages; thus, they test the hypotheses for C++ and Java classes separately. The results showed the usefulness of CBO in C++ projects.

Janes et al. (2006) performed three regression techniques on five real-time telecommunication system. The objective was to assess the performance of CK metric suite in fault prediction. They reported the statistically significant performance of RFC in all the projects, while CBO was found useful on some of the analyzed projects.

Olague et al. (2007) empirically evaluated three object-oriented metric suites (CK, MOOD, and QMOOD) in predicting faults on six Rhino versions. Using bivariate correlation between metrics and faults they concluded RFC as strongly correlated with software faults, while CBO has minor to moderate correlation. Next, by using logistic regression analysis, RFC was found significant in all six versions of Rhino, whereas CBO was found significant in five versions of Rhino.

Xu, Ho & Capretz (2008) assessed the usefulness of CBO and RFC on NASA’s KC1 dataset and conclude the effectiveness of both metrics using Correlation and Regression analyses. However, their third experiment using Neuro-fuzzy approach resulted against the effectiveness of both metrics.

Zimmermann & Nagappan (2008) assessed the dependency factor in predicting *fp* binaries in Windows Server 2003. The dependency factor includes call dependencies, data dependencies, and dependencies specific to Windows. Binary refers to Portable executables, COMs, or DLLs. Call dependency includes import calls, export calls. The dataset comprised 2252 binaries. A dependency graph was generated using MaX and fault data was collected using the post-release fault archive maintained by Microsoft. The prediction was done using classification and ranking (number of faults). They evaluated CCM, Nagappan’s CyclicClassCoupling, Fan-in, and Fan-out along with some non-coupling metrics.

Kpodjedo et al. (2009) investigated the fault predictive ability of CK metric suite and their proposed ECGM metrics. In addition to ECGM, the most accurate model was the one, which was built on CBO and RFC inclusively.

English et al. (2009) evaluated the usefulness of CK metric suite using Bugzilla reports and CVS commits of two software products Eclipse JDT and Mozilla. The authors used univariate and multivariate logistic regression to assess the impact of individual metrics and LOC with software faults. They reported high correctness values of RFC and CBO. Next, in linear regression modeling, RFC and CBO were found reasonable predictors of software faults. Finally, they gave a verdict that LOC along with CBO and RFC are the best predictors of *fp* classes.

Jureczko & Spinellis (2010) developed a regression model for predicting faults using CK metric suite and LOC. They used five proprietary and eleven open-source projects. In the process of eliminating the least correlated metrics, they dropped RFC, while keeping CBO.

Shatnawi (2010) investigated the acceptable risk level using CK metric suite. Two versions of Eclipse 2.0 and 2.1 were taken as case studies. Modeling was done through univariate logistic regression. CBO and RFC were found significant predictors of faults at the 95% confidence level.

Rathore & Gupta (2012) evaluated 19 class level metrics (including coupling metrics) on five publicly available project datasets. The authors first evaluated each metric independently using univariate logistic regression. Next, the correlation between metrics was computed, where strongly correlated metrics were dropped and the remaining subset of metrics was evaluated using multiple releases of the same software. In their first experiment, they concluded that CBO, RFC, import, and export coupling metrics are significantly correlated with software fault in four datasets.

He et al. (2015) aimed to build simplified metric set for SFP. They took 34 releases of 10 open-source projects from PROMISE repository. Model building was done using J48, LR, NB, DT, SVM, and BN. Independent variables were CBO, RFC, Ca, Ce, and CBM and the dependent variable was Binary. They first selected TOPK metrics for their experiment, wherein CBO, RFC, and Ce were selected.

Kumari & Rajnish (2015) proposed a class level complexity metric (CLCM). Their objective was to evaluate the performance difference of CLCM and some other coupling metrics CBO, RFC, MPC, LMC, Fan-out, and EXT. Dataset was collected from three versions of Eclipse 2.0, 2.1, and 3.0. The experiment was performed on each version independently. Binary (*fp* and *nfp*) and multilabel (severity level - Minimum, Low, Medium, and High) classifications were performed. For both types of dependent variables, Spearman correlation coefficient and univariate logistic regression were used to investigate the impact of a metric on SFP. The results of this experiment showed the strong correlation of coupling metrics with faults and classification accuracy for all coupling metrics laid between 0.70 to 0.75. More specifically EXT, MPC, and RFC had the strongest impact on pre-release faults.

Kumar, Tirkey & Rath (2018) performed an experiment to predict the presence and absence of fault. The independent variables used in the experiment were CBO, RFC, Ce, Ca, CBM, WMC, DIT, NOC, LCOM, NPM, LOC, LCOM3, DAM, MOA, MFA, IC, CAM, AMC, Max-CC, and Avg-CC. The experiment was performed on 31 projects developed in Java. The authors applied Chi-squared test, Gain ratio feature evaluation, OneR, Feature evaluation, Univariate Logistic regression, and Principal component analysis. Their result concluded a strong association of coupling metrics with software faults.

Rizwan, Nadeem & Sindhu (2020a) is the most recent study that evaluated the exclusive impact of combined coupling metrics in SFP. The authors evaluated seven coupling metrics on 87 different publicly available datasets. Dataset were split with the wrapper technique. Resulting in 474 split datasets are used for the experiments. Support Vector Machine was used for modeling and performance evaluation was computed using entropy-loss. They reported that the set {CBO, DC, Fan-in} has outperformed the rest of the 30 feature set. Finally, through their novel metrics ranking mechanism, Ce has the highest score.

Table 2 summarizes the included studies. The included studies answer first of our two research question. The studies depict that, Coupling metrics in general, and CBO, RFC, Fan-in, and Fan-out are specifically found useful in predicting software faults, irrespective of the dataset size, type of dependent variable. However, the most recent study in the theoretical evaluation of coupling metrics conducted by Rizwan, Nadeem & Sindhu (2020b) reported that the difference between coupling levels (Myers, 1975; Yourdon & Constantine, 1979, Page-Jones, 1988) has been ignored by most of the metrics. Table 3 shows the summary of these facts.

**Table 2:** Catagorization of studies w.r.t type of dependent variables used in the studies.

Study	Coupling metrics	Non-coupling metrics	Dependent variable
(Briand et al., 1998)	CBO, RFC, MPC, ICP, NIHICP, DAC, OCAEC, OCMEC, OMMEC, OMMIC, OCAIC, OCMIC, IFCAIC, IFCMIC, IFMMIC, FCAEC, FCMEC	NMO, SIX, NMA, LOC, WMC, DIT, AID, NOA, NOP, NMI, NOC, NOD, CLD, ACAIC, DCAEC, ACMIC, DCMEC, AMMIC, DMMEC	Binary
(El Emam et al., 1999)	CBO, RFC, OCAEC, OCMEC, OMMEC, OMMIC, OCAIC, OCMIC, IFCAIC, IFCMIC, IFMMIC, FCAEC, FCMEC	LCOM, SLOC, WMC, DIT, ACAIC, DCAEC, ACMIC, DCMEC, AMMIC, DMMEC	Binary
(Briand et al., 2000)	CBO, RFC, MPC, ICP, NIHICP, DAC, OCAEC, OCMEC, OMMEC, OMMIC, OCAIC, OCMIC, IFCAIC, IFCMIC, IFMMIC, FCAEC, FCMEC	NMO, SIX, NMA, LOC, WMC, DIT, AID, NOA, NOP, NMI, NOC, NOD, CLD, ACAIC, DCAEC, ACMIC, DCMEC, AMMIC, DMMEC	Binary
(El Emam et al., 2001)	CBO, RFC, OCAEC, OCMEC, OMMEC, OMMIC, OCAIC, OCMIC, IFCAIC, IFCMIC, IFMMIC, FCAEC, FCMEC, FMMEC, NPAVG	SIX, LCOM, SLOC, WMC, DIT, ACAIC, DCAEC, ACMIC, DCMEC, AMMIC, DMMEC, NMA, NMO	Binary
(Shatnawi, Li & Zhang, 2006)	CTA, CTM, CBO, RFC	WMC, DIT, NOC, NOAM, NOOM, NOA, NOO	Binary
(Aggarwal et al., 2007)	CBO, RFC, FCAEC, FCMEC, FMMEC, IFCAIC, IFCMIC, IFMMIC, OCAEC, OCMEC, OMMEC, OMMIC, OCAIC	LCOM1, LCOM2, NOC, DIT, WMC, ACAIC, DCAEC, ACMIC, DCMIC, DCMEC, AMMIC, DMMEC, LOC	Binary
(Aggarwal et al., 2009)	CBO, RFC, DAC, MPC, ICP, NIHICP, FCAEC, FCMEC, FMMEC, IFCAIC, IFCMIC, IFMMIC, OCAEC, OCAIC, OCMIC, OCMEC, OMMEC, OMMIC	IHICP, ACAIC, DCAEC, ACMIC, DCMEC, AMMIC, DMMEC, LCOM1, LCOM2, LCOM3, TCC, LCC, ICH, NOC, DIT, CLD, NOP, NOD, NOA, NMO, NMI, NMA, SIX, AID, NA, NM, WMC, PM, NPM, NPARA, LOC	Binary
(English et al., 2009)	CBO, RFC	WMC, DIT, NOC, LOC	Binary
(Malhotra, Kaur & Singh, 2010)	CBO, RFC	WMC, DIT, NOC, LCOM, SLOC	Binary
(Jureczko & Spinellis, 2010)	CBO, RFC, Ca, Ce	CBM, WMC, DIT, NOC, LCOM, LCOM3, NPM, DAM, MOA, MFA, CAM, IC, AMC, CC, LOC	Binary
(Shatnawi, 2010)	CBO, RFC	WMC, DIT, NOC	Binary
(Rathore & Gupta, 2012)	CBO, RFC, Ca, Ce,	WMC, DIT, NOC, IC, CBM, MFA, LCOM, LCOM3, CAM, MOA, NPM, DAM, AMC, LOC, CC	Binary
(He et al., 2015)	RFC, CBO, Ca, Ce	CBM, WMC, DIT, LCOM, NOC, DAM, NPM, MFA, CAM, MOA, IC, AMC, LCOM3, MAX CC, AVG CC, LOC	Binary
(Gyimothy, Ferenc & Siket, 2005)	RFC, CBO,	WMC, DIT, LOC, LCOM, NOC, LCOMN	Binary and Numerical
(Zimmermann & Nagappan, 2008)	Fan-in, Fan-out	LOC, No. of parameters, CC, NOM, SubClasses DIT, ClassCoupling, CCC	Binary and Numerical
(Kpodjedo et al., 2009)	CBO, RFC	WMC, DIT, NOC, LCOM, EC, CR, LOC	Binary and Numerical
(Kumari & Rajnish, 2015)	RFC, MPC, CBO	NOS, UWCS, CC, NLOC, EXT, LMC, TCC, PACK, NOM, LOM2, INST, MAXCC, FOUT, AVCC, CLCM	Binary, Multinomial
(Rizwan, Nadeem & Sindhu, 2020a)	CBO, RFC, Ce, Ca, Fan-in, Fan-out	None	Nominal
(Kumar, Tirkey & Rath, 2018)	CBO, RFC, Ce, Ca	CBM, WMC, DIT, NOC, LCOM, NPM, LOC, LCOM3, DAM, MOA, MFA, IC, CAM, AMC, Max-CC, Avg-CC	Nominal
(Johari & Kaur, 2012)	CBO, RFC	WMC, DIT, NOC, LCOM, Token count, WMC(CC)	Numerical (Bug count and Revision count)
(Troy & Zweben, 1981)	X[1-7, 19-21]^1^	X [8 - 18]	Numerical
(Kitchenham, Pickard & Linkman, 1990)	Fan-in, Fan-out	LoC, CC	Numerical
(Binkley & Schach, 1997b)	CBO, NSSR, NCC, CDM, Fan-in, Fan-out, RFC	LoC, WMC, DIT, CHNL, NOC, NOD, NCIM, WIH, HIH	Numerical
(Binkley & Schach, 1997a)	Fan-in, Fan-out, CDM, OSC	CC, LoC	Numerical
(Binkley & Schach, 1998a, 1998b)	Fan-in, Fan-out, CDM, OSC	CC, LoC	Numerical
(Binkley & Schach, 1998a, 1998b)	Fan-in, Fan-out, CBO, NCC, NSSR, CDM, RFC	WMC, DIT, CHNL, NOC, NCIM, WIH, HIH, CC, LOC, NOD, No. of global variables, No. of clients	Numerical
(Harrison, Counsell & Nithi, 1998)	CBO, NAS	None	Numerical
(Tang, Kao & Chen, 1999)	CBO, RFC	DIT, NOC, WMC, IC, CBM, NOMA, AMC, IC, CBM, NOMA, AMC	Numerical
(Subramanyam & Krishnan, 2003)	CBO, RFC	DIT, LCOM, NOC, NOM, SLOC	Numerical
(Janes et al., 2006)	CBO, RFC	DIT, LCOM, NOC, NOM, SLOC	Numerical
(Abubakar, AlGhamdi & Ahmed, 2006)	CBO, RFC, Fan-in	PPD, ATPD, CBO, DIT, LCOM, NOC, RFC, WMPC, and DOC	Numerical
(Olague et al., 2007)	CBO, RFC	DIT, LCOM, NOC, WMC, MC, AHF, AIF, MHF, MIF, CIS, DAM, DCC, MFA	Numerical
(Xu, Ho & Capretz, 2008)	CBO, RFC	WMC, DIT, NOC, SLOC, LCOM	Numerical
(Elish, Al-Yafei & Al-Mulhem, 2011)	Ca, Ce, CBO, RFC	NC, I, D, AHF, MHF, AIF, MIF, CF, PF, WMC, LCOM, DIT, NOC	Numerical
(Anwer et al., 2017)	Ca, Ce, CBO	None	Numerical
(Shatnawi & Li, 2008)	CTA, CTM, CBO, RFC	WMC, DIT, NOC, NOAM, NOOM, NOA, NOO	Ordinal (Severity)

**Note:**

1 Detail of Xs may be find from respective article.

**Table 3:** Coupling metrics w.r.t. levels’ and principles’ coverage.

		Fan-in	Fan-out	CBO	RFC	CCM	DAC	MPC	NIHICP	ICP	Ca	Ce	I	CDM	OSMC	Briandsuite
Levels	Content	○	○	○	○	○	○	○	○	○	○	○	○	○	○	○
	Common	○	○	○	○	○	○	◐	○	○	○	○	○	○	●	○
	Control	◐	◐	○	◐	◐	○	◐	◐	◐	◐	◐	◐	○	●	◐
	Descriptive	◐	◐	◐	◐	◐	○	◐	◐	◐	◐	◐	◐	○	●	◐
	Stamp	◐	◐	◐	◐	◐	○	◐	◐	◐	◐	◐	◐	○	●	◐
	Data	◐	◐	◐	◐	◐	○	◐	◐	◐	◐	◐	◐	○	●	◐
	Zeroscale	◐	◐	◐	◐	◐	○	◐	◐	◐	◐	◐	◐	○	○	○
Principles	Broad	✗	✗	✗	✗	✗	✗	✗	✓	✓	✗	✗	✗	✗	✗	✗
	Hidden	✗	✗	✗	✗	✗	✗	✗	✗	✗	✗	✗	✗	✗	✗	✗
	Rigid	✗	✗	✗	✗	✗	✗	✗	✗	✗	✗	✗	✗	✗	✗	✗

This discussion collectively spurs the derivation of new coupling metrics that provide wider coverage of the coupling levels and important coupling factors, thus are expected to be a good prediction of software faults. The following sections are dedicated for the derivation and evaluation of such metric.

## Vovel Metrics: Improved Coupling Metrics

Keeping in view the importance of volume of data flow and levels of coupling, we propose two novel coupling metrics named; *Vovel-in* and *Vovel-out* for inner and outer coupling respectively. The term *Vovel* has been made from the first two characters of word Volume and the last three characters from the word level. This section elaborates the process of deriving/computing the proposed Vovel metrics.

### Derivation of vovel metrics

**Figure 1:**
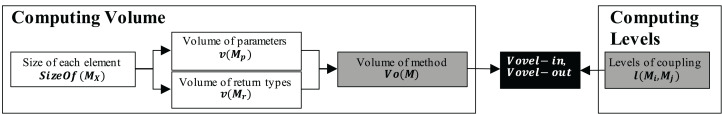
Process of deriving Vovel metrics.

The derivation of Vovel metrics constitutes two important factors, i.e., Volume of data flow and levels of coupling. Figure 1 illustrates the components and composition of the Vovel metrics.

#### Computing volume of each method

Volume refers to the amount of data flow between modules, which is usually done through parameters and/or return values in case of the methods. Amongst the existing coupling metrics, GIF (Henry & Kafura, 1981), LIF (Henry & Kafura, 1981), DataC, SC, ICP, and NIHICP consider volume. Likewise, the dependency relationship covered by CSSM metric (Singh & Singh, 2014) considers the parameters and return types. However, these metrics consider only the number of parameters, whereas, volume is not solely dependent on the number of parameters but the nature of parameters as well. Like, primitive data types are relatively narrow in carrying information as compared to arrays. Therefore, the volume addressed by these coupling metrics could not satisfy the due coverage of volume of information flow. Therefore, we use a novel approach for computing volume, which is shown in Eq. (1).

(1) }{}Vol(M) = \left\{{\matrix {{\hskip-60pt}1,  {For\; content\; coupling} \cr {\hskip-41pt} v(M_{c}),  {For\; content\; coupling}\cr vr(M_{p})+v(M_{r}),  {\hskip-46pt}\text{otherwise} }}  \right.

where,

*M*_*p*_ is a list of parameters in method *M* , and *v*(*M*_*p*_) shows the volume of method *M* w.r.t. its parameters. It is computed using Eq. (2)

*M*_*r*_ is a list of return types in method *M* , and *v*(*M*_*r*_) shows the volume of method *M* w.r.t. its return types. It is computed using Eq. (2)

*M*_*c*_ is a list of common variable types that a method *M* reads or writes, and *v*(*M*_*c*_) shows the volume of the shared variables. It is computed using Eq. (2)

We assigned a weight of ‘1’ for content coupling, since there is no significant flow of information in this coupling type.

The *v*(*M*_*r*_) covers the languages that allow more than one value to be returned. One such language is Python. All *v*(*M*_*p*_), *v*(*M*_*r*_), and *v*(*M*_*c*_) are computed by Eq. (2).

(2) }{}v(M_{x})= \left\{\matrix {\sum{_{j=1}^{n}} SizeOf\;(M_{{X}_{j}}),  {\hskip-18pt} n \gt 0 \cr {\hskip-70pt} 1,  \text{otherwise}\\ } \right.

where, *SizeOf*(*M*_*Xj*_) shows the memory allocated to an element *j* from the list of parameter/return/common variable type *X*. Equation (2) considers memory allocated at the time of declaration, however, the memory which is allocated at runtime is beyond the scope of this study.

#### Inducing coupling levels

Couplings can vary in strength w.r.t. the levels. Rizwan, Nadeem & Sindhu (2020b) provide a list of 10 coupling levels. The proposed metrics include all the levels concluded by Rizwan et al. and assign weights using function as per their strength reported by them (see Eq. (3)).

(3) }{}l(M_{i}, M_{j})= \left\{{\matrix { 0,  {\hskip-56pt} No\;coupling\;of\;M_{i}\;to\;M_{j}\cr 1,  {\hskip-28pt} Zero\;scale\;coupling\;of\;M_{i}\;to\;M_{j}\cr 2,  {\hskip-48pt} Data\;coupling\;of\;M_{i}\;to\;M_{j}\cr 3,  {\hskip-42pt} Stamp\;coupling\;of\;M_{i}\;to\;M_{j}\cr 4,  Scalar \;descriptive\;coupling\;of\;M_{i}\;to\;M_{j}\cr 5,  Stamp\;descriptive\;coupling\;of\;M_{i}\;to\;M_{j}\cr 6,  {\hskip-52pt} Scalar \;control \;of\;M_{i}\;to\;M_{j}\cr 7,  {\hskip-50pt} Stamp\;control \;of\;M_{i}\;to\;M_{j}\cr 8,  {\hskip-24pt} Scalar\;common\;between\;M_{i}\;to\;M_{j}\cr 9,  {\hskip-24pt} Stamp\;common\;between\;M_{i}\;to\;M_{j}\cr 10,  {\hskip-40pt} Content\;coupling\;of\;M_{i}\;to\;M_{j} }} \right.

where, *l*(*M*_*i*_, *M*_*j*_) represents level of coupling from method *M*_*i*_ to *M*_*j*_. *No coupling* is assigned zero weight, which shows that there is no coupling between the modules, in fact only the control is being transferred from one module to another. This level helps us to simplify the metrics’ equation.

#### Combining coupling levels and volume of data flow

Since, the coupling levels are directional (except common coupling), we derive two metrics *Vovel-in* and *Vovel-out* to accommodate two distinct directions. These two metrics are computed by combining the function *l*(*M*_*i*_, *M*_*j*_) and Eq. (1). The *Vovel-in* and *Vovel-out* of a method *M* can be computed by Eqs. (4) and (5), respectively.

(4) }{}Vovel-out(M)=Vol(M) \times \sum_{j=1}^{m} l (M_{j}, M)

(5) }{}Vovel-out(M)=\sum_{j=1}^{m} l (M, M_{i}) \times Vol(M_{j})

where, *m* is the number of all the methods in the software product excluding *M* . The equations compute coupling of a **method** with other methods. However, the equations can slightly be modified to Eqs. (6) and (7) to compute the coupling of a **class** with other classes.

(6) }{}Vovel-out(C)=\sum_{j=1}^{n} \sum_{i=1}^{m} l (M_{j}, M_{i}) \times Vol(M_{i})

(7) }{}Vovel-out(C)=\sum_{i=1}^{n} \sum_{j=1}^{m} l (M_{i}, M_{j}) \times Vol(M_{j})

where, *n* is the number of methods in class *C* and *m* is the number of all the methods belonging to other classes. In the Eqs. (4), (5), (6), and (7) the volume of a called method is computed.

### Demonstration of vovel metrics computation

In this section, we demonstrate the computation of Vovel metrics. We take eight hypothetical Java methods and their different signatures to demonstrate the computation of volume of methods. Table 4 illustrates the methods and the volumes associated with each method in bits^1^ 1We took ‘bit’ instead of higher unit, for being it more discriminating..

**Table 4:** Sample Java based methods and their volume.

Coupled component	*v*( *M*_*r*_)	*v*(*M*_*p*_)	*v*(*M*_*c*_)	*Vol*(*M*)
void **A**()	0	0	–	0
void **B**(int)	0	32	–	32
void **C**(boolean, short)	0	1 + 16	–	17
void **D**(float, char, bool)	0	32 + 16 + 1	–	49
int **E**()	32	0	–	32
char **F**(boolean)	16	1	–	17
boolean **G**(double, int)	1	64 + 32	–	97
int **H**(int, char, object)	32	32 + 16 + 1	–	81
int **C**	–	–	32	32

In Table 4 we assign 16-bit size to the object since it is the minimum object size for modern 64-bit JDK object. However, in reality, we consider the memory allocated to an object, which is implementation-dependent, so it may be equal to or greater than 16. Finally boxed types, arrays, Strings, and other containers like multidimensional arrays, memory allocated are implementation-dependent. In Java one way to get an estimate of these container sizes is to implement Instrumentation interface (Java, 2018).

**Figure 2:**
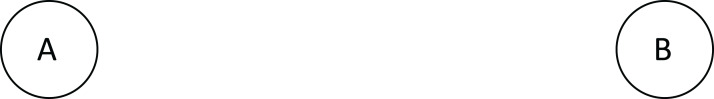
Methods are not calling each other.

**Figure 3:**
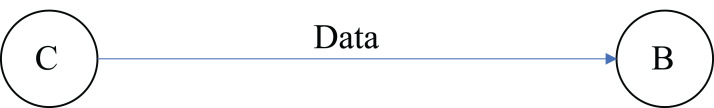
Two methods are data coupled.

**Figure 4:**
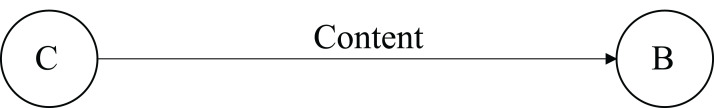
Two methods are content coupled.

**Figure 5:**
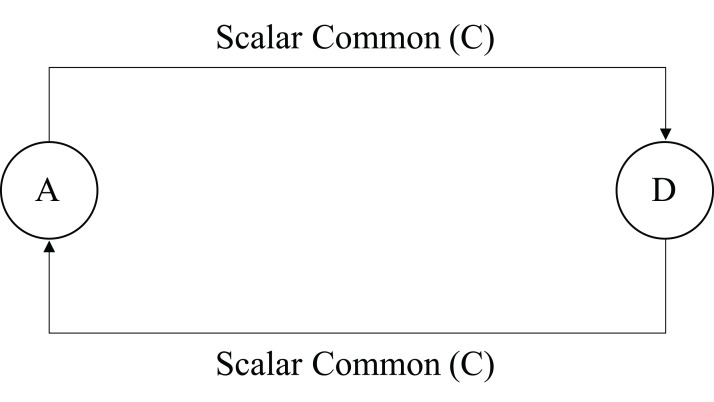
Two methods are scalar common coupled.

**Figure 6:**
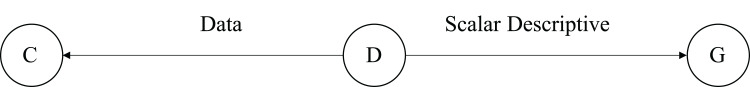
A method is data coupled with one method and scalar coupled with another method.

**Figure 7:**
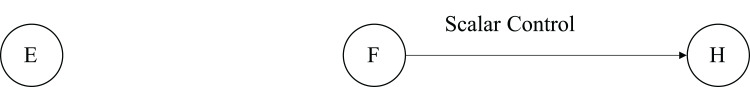
Two methods are scalar coupled while third method is isolated.

**Figure 8:**
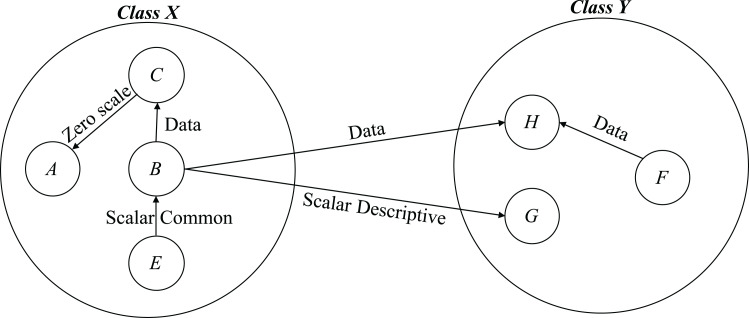
Example for computing Vovel metrics at class levels.

These eight methods are used in Figs. 2 to 7 to compute Vovel metric at method level and in Fig. 8 to compute the Vovel metrics of a class.

From Figs. 2 to 8, methods are denoted by a circle with the name inside it. The arrow is directed from the caller to called method. The label on the arrow shows level of coupling between the methods on either side of an arrow.

*Vovel* − *in*(*A*) = *Vol*(*A*) × *l*(*B*, *A*) = 0 × 0 = 0

*Vovel* − *in*(*B*) = *Vol*(*B*) × *l*(*A*, *B*) = 32 × 0 = 0

*Vovel* − *out*(*A*) = *Vol*(*B*) × *l*(*A*, *B*) = 32 × 0 = 0

*Vovel* − *out*(*B*) = *Vol*(*A*) × *l*(*B*, *A*) = 0 × 0 = 0

*Vovel* − *in*(*B*) = *Vol*(*B*) × *l*(*C*, *B*) = 32 × 2 = 64

*Vovel* − *in*(*C*) = *Vol*(*C*) × *l*(*B*, *C*) = 17 × 0 = 0

*Vovel* − *out*(*B*) = *Vol*(*C*) × *l*(*B*, *C*) = 17 × 0 = 0

*Vovel* − *out*(*C*) = *Vol*(*B*) × *l*(*C*, *B*) = 32 × 2 = 64

*Vovel* − *in*(*B*) = *Vol*(*B*) × *l*(*C*, *B*) = 1 × 10 = 10

*Vovel* − *in*(*C*) = *Vol*(*C*) × *l*(*B*, *C*) = 17 × 0 = 0

*Vovel* − *out*(*B*) = *Vol*(*C*) × *l*(*B*, *C*) = 17 × 0 = 0

*Vovel* − *out*(*C*) = *Vol*(*B*) × *l*(*C*, *B*) = 1 × 10 = 10

*Vovel* − *in*(*A*) = *Vol*(*A*) × *l*(*D*, *A*) = 32 × 8 = 256

*Vovel* − *in*(*D*) = *Vol*(*D*) × *l*(*A*, *D*) = 32 × 8 = 256

*Vovel* − *out*(*A*) = *Vol*(*D*) × *l*(*A*, *D*) = 32 × 8 = 256

*Vovel* − *out*(*D*) = *Vol*(*A*) × *l*(*D*, *A*) = 32 × 8 = 256

*Vovel* − *in*(*G*) = *Vol*(*G*) × *l*(*C*, *G*) + *Vol*(*G*) × *l*(*D*, *G*) = 97 × 0 + 97 × 4 = 388

*Vovel* − *in*(*C*) = *Vol*(*C*) × *l*(*G*, *C*) + *Vol*(*C*) × *l*(*D*, *C*) = 17 × 0 + 17 × 2 = 34

*Vovel* − *in*(*D*) = *Vol*(*D*) × *l*(*G*, *D*) + *Vol*(*D*) × *l*(*C*, *D*) = 49 × 0 + 49 × 0 = 0

*Vovel* − *out*(*G*) = *Vol*(*C*) × *l*(*G*, *C*) + *Vol*(*D*) × *l*(*G*, *D*) = 17 × 0 + 49 × 0 = 0

*Vovel* − *out*(*C*) = *Vol*(*G*) × *l*(*C*, *G*) + *Vol*(*D*) × *l*(*C*, *D*) = 97 × 0 + 49 × 0 = 0

*Vovel* − *out*(*D*) = *Vol*(*G*) × *l*(*D*, *G*) + *Vol*(*C*) × *l*(*D*, *C*) = 97 × 4 + 17 × 2 = 422

*Vovel* − *in*(*E*) = *Vol*(*E*) × *l*(*F*, *E*) + *Vol*(*E*) × *l*(*H*, *E*) = 32 × 0 + 32 × 0 = 0

*Vovel* − *in*(*F*) = *Vol*(*F*) × *l*(*E*, *F*) + *Vol*(*F*) × *l*(*H*, *F*) = 17 × 0 + 17 × 0 = 0

*Vovel* − *in*(*H*) = *Vol*(*H*) × *l*(*E*, *H*) + *Vol*(*H*) × *l*(*F*, *H*) = 81 × 0 + 81 × 6 = 486

*Vovel* − *out*(*E*) = *Vol*(*F*) × *l*(*E*, *F*) + *Vol*(*H*) × *l*(*E*, *H*) = 17 × 0 + 81 × 0 = 0

*Vovel* − *out*(*F*) = *Vol*(*E*) × *l*(*F*, *E*) + *Vol*(*H*) × *l*(*F*, *H*) = 32 × 0 + 81 × 6 = 486

*Vovel* − *out*(*H*) = *Vol*(*E*) × *l*(*H*, *E*) + *Vol*(*F*) × *l*(*H*, *F*) = 32 × 0 + 17 × 0 = 0

Figure 8 illustrates the computation of Vovel metric at class level. Figure contains two classes X and Y. Class X contains four methods A, B, C, and E, whereas class Y contains three methods F, G, and H.

**Computing Vovel metrics for Class X:**

*Vovel* − *in*(*A*) = *Vol*(*A*) × *l*(*F*, *A*) + *Vol*(*A*) × *l*(*G*, *A*) + *Vol*(*A*) × *l*(*H*, *A*) = 0 × 0 + 0 × 0 + 0 × 0 = 0

*Vovel* − *in*(*C*) = *Vol*(*C*) × *l*(*F*, *C*) + *Vol*(*C*) × *l*(*G*, *C*) + *Vol*(*C*) × *l*(*H*, *C*) = 17 × 0 + 17 × 0 + 17 × 0 = 0

*Vovel* − *in*(*B*) = *Vol*(*B*) × *l*(*F*, *B*) + *Vol*(*B*) × *l*(*G*, *B*) + *Vol*(*B*) × *l*(*H*, *B*) = 32 × 0 + 32 × 0 + 32 × 0 = 0

*Vovel* − *in*(*E*) = *Vol*(*E*) × *l*(*F*, *E*) + *Vol*(*E*) × *l*(*G*, *E*) + *Vol*(*E*) × *l*(*H*, *E*) = 32 × 0 + 32 × 0 + 32 × 0 = 0

*Vovel* − *in*(*X*) = *Vovel* − *in*(*A*) + *Vovel* − *in*(*C*) + *Vovel* − *in*(*B*) + *Vovel* − *in*(*E*) = 0 + 0 + 0 + 0 = 0

*Vovel* − *out*(*A*) = *Vol*(*F*) × *l*(*A*, *F*) + *Vol*(*G*) × *l*(*A*, *G*) + *Vol*(*H*) × *l*(*A*, *H*) = 17 × 0 + 97 × 0 + 81 × 0 = 0

*Vovel* − *out*(*C*) = *Vol*(*F*) × *l*(*C*, *F*) + *Vol*(*G*) × *l*(*C*, *G*) + *Vol*(*H*) × *l*(*C*, *H*) = 17 × 0 + 97 × 0 + 81 × 0 = 0

*Vovel* − *out*(*B*) = *Vol*(*F*) × *l*(*B*, *F*) + *Vol*(*G*) × *l*(*B*, *G*) + *Vol*(*H*) × *l*(*B*, *H*) = 17 × 0 + 97 × 4 + 81 × 2 = 550

*Vovel* − *out*(*E*) = *Vol*(*F*) × *l*(*E*, *F*) + *Vol*(*G*) × *l*(*E*, *G*) + *Vol*(*H*) × *l*(*E*, *H*) = 17 × 0 + 97 × 0 + 81 × 0 = 0

*Vovel* − *out*(*X*) = *Vovel* − *out*(*A*) + *Vovel* − *out*(*C*) + *Vovel* − *out*(*B*) + *Vovel* − *out*(*E*) = 0 + 0 + 550 + 0 = 550

**Computing Vovel metrics for Class Y:**

*Vovel-in(F) = Vol(F) × l(A,F) + Vol(F) × l(B,F) + Vol(F) × l(C,F) + Vol(F) × l(E,F)* = 17 × 0 + 17 × 0 + 17 × 0 + 17 × 0 = 0

*Vovel-in(G) = Vol(G) × l(A,G) + Vol(G) × l(B,G) + Vol(G) × l(C,G) + Vol(G) × l(E,G)*= 97 × 0 + 97 × 4 + 97 × 0 + 97 × 0 = 388

*Vovel-in(H) = Vol(H) × l(A,H) + Vol(H) × l(B,H) + Vol(H) × l(C,H) + Vol(H) × l(E,H)*= 81 × 0 + 81 × 2 + 81 × 0 + 81 × 0 = 162

*Vovel-in(Y) = Vovel-in(F) + Vovel-in(G) + Vovel-in(H)*= 0 + 388 + 162 = 550

*Vovel-out(F) = Vol(A) × l(F,A) + Vol(B) × l(F,B) + Vol(C) × l(F,C) + Vol(E) × l(F,E)* = 0 × 0 + 32 × 0 + 17 × 0 + 32 × 0 = 0

*Vovel-out(G) = Vol(A) × l(G,A) + Vol(B) × l(G,B) + Vol(C) × l(G,C) + Vol(E) × l(G,E)* = 0 × 0 + 32 × 0 + 17 × 0 + 32 × 0 = 0

*Vovel-out(H) = Vol(A) × l(H,A) + Vol(B) × l(H,B) + Vol(C) × l(H,C) + Vol(E) × l(H,E)* = 0 × 0 + 32 × 0 + 17 × 0 + 32 × 0 = 0

*Vovel-out(Y) = Vovel-out(F) + Vovel-out(G) + Vovel-out(H)* = 0 + 0 + 0 = 0

### Significance of vovel metrics

The proposed metrics have some unique significance also,

1. The metrics accommodate both structural and OO paradigm.

2. Some programming languages do not support multiple values to be returned, while some others do. However, the metrics support both types of languages.

3. Numerous coupling levels proposed by the community (Rizwan, Nadeem & Sindhu, 2020b) can be accommodated by just modifying the function *l*(*M*_*i*_, *M*_*j*_). Hence, the Vovel metrics are flexible enough to accommodate the difference in numbers of coupling levels and diversity of coupling levels’ placement.

## Materials and Methods

### Case study

The proposed metrics need to be validated empirically for viability. D’Ambros, Lanza & Robbes (2010) develop fault datasets of five projects; Eclipse JDT Core 3.4 (www.eclipse.org/jdt/core/), Equinox framework 3.4 (www.eclipse.org/equinox/), Apache Lucene 2.4 (lucene.apache.org), Mylyn 3.1 (www.eclipse.org/mylyn/), and Eclipse PDE UI 3.4.1 (www.eclipse.org/pde/pde-ui/). These projects are developed in Java. These projects are public, which include fault information as well.

Tóth, Gyimesi & Ferenc (2016) compute numerous software product metrics from the selected five projects. Out of these metrics, we selected four coupling metrics {CBO, Fan-in, Fan-out, RFC} because of their reported effectiveness by SFP community (English et al., 2009; Kumar, Tirkey & Rath, 2018; Rizwan, Nadeem & Sindhu, 2020a). However, we computed the two proposed metrics{Vovel-in, Vovel-out} using Javaparser (Parser, 2017). Javaparser contains a set of libraries implementing a Java 1.0 to analyse and parse the Java projects. It is used by some other authors also (Anquetil, 2013; Tufano et al., 2018b, 2018a). The statistical description of the metrics in all five dataset are shown in Table 5.

**Table 5:** Statistical description of metrics in the selected datasets.

Datasets	Parameters	Ce	CBO	RFC	Fan-in	Fan-out	Vovel-in	Vovel-out
**lucene**	Mean	5.4	6.9	18.5	4.4	5.5	344.1	547.6
	Std	5.4	7.6	23.4	12.1	6.8	2,003	2,983
	Min	0	0	1	0	0	0	0
	25%	1	2	6	1	2	0	21
	50%	6	4	12	1	3	0	129
	75%	7	9	23	4	7	87	423
	Max	81	64	308	174	67	57,776	84,187
**Eclipse JDT**	Mean	12	14.5	37	5.4	7.4	503.6	413.3
	Std	17	19.4	55.9	13.7	9.7	2,259.1	566.9
	Min	0	0	0	0	0	0	0
	25%	2	4	9	1	2	0	86.8
	50%	7	9	20	2	4	25.8	296.3
	75%	20	18	42	4	10	243	539
	Max	300	214	600	137	93	30,181.3	9,041.5
**PDE UI**	Mean	5.3	6.6	16.9	4.1	5.8	348.4	416.2
	Std	5.2	7.6	20.7	13.4	6.8	1,846.5	1,710.3
	Min	0	0	1	0	0	0	0
	25%	4	2	5	1	1	0	21
	50%	6	4	10	1	4	0	129
	75%	7	9	21	3	8	84.5	400.5
	Max	110	80	308	355	67	57,776	84,187
**Equinox**	Mean	10.3	6.6	19.8	3.4	8.4	544.2	945.7
	Std	9	8.3	27.9	5	10	3,034	5,034.5
	Min	0	0	1	0	0	0	0
	25%	2.7	1.5	5	1	2	0	0
	50%	10	5	11	2	5	0	131
	75%	15	8	23	4	11	158	678
	Max	105	56	213	32	67	57,776	84,187
**Mylyn**	Mean	9	6.1	16.2	4.4	5.2	315.5	431.6
	Std	6.4	7.2	21	13.8	6.5	1,694.2	2,091.8
	Min	0	0	1	0	0	0	0
	25%	6	1	5	1	1	0	18
	50%	7	4	10	1	3	0	119
	75%	9	8	20	3	7	70	374
	Max	94	80	308	223	67	57,776	84,187

**Figure 9:**
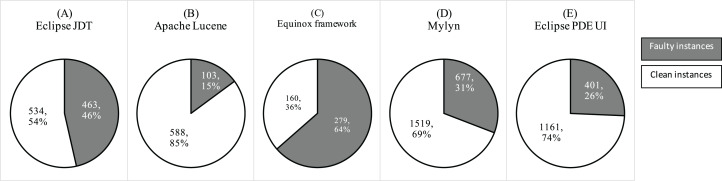
Ratio of faulty and clean instances in the selected datasets.

The dichotomous dependent variables that we used in our study are *fp* and *nfp*. Toth et al. assigned numerical labels using bug tracking system (Tóth, Gyimesi & Ferenc, 2016). We rely on their labels. However, we convert the numerical bug label to dichotomous variable by converting 0 bugs to *nfp*, and *fp* otherwise. Figure 9 shows the fault ratio in the selected projects.

### Methodology

We first perform the ULR to compute the significance of the coupling metric. The significant metrics are later accessed for the presence of association with the Vovel metrics using Spearman correlation coefficient. Later the least correlated metrics are used to build multivariable logistic regression model. The methodology is followed by other studies also (Briand et al., 1998; Tang, Kao & Chen, 1999; Gyimothy, Ferenc & Siket, 2005; Shatnawi, Li & Zhang, 2006; Aggarwal et al., 2007; Olague et al., 2007; Xu, Ho & Capretz, 2008; Shatnawi & Li, 2008; Aggarwal et al., 2009; English et al., 2009; Shatnawi, 2010; Johari & Kaur, 2012; Rathore & Gupta, 2012; Kumari & Rajnish, 2015; Kumar, Tirkey & Rath, 2018). Since our dataset are skewed, ULR and MLR are good choice. The reason is that these algorithms are least susceptible to imbalance dataset (Luque et al., 2019).

#### Univariate logistic regression

Logistic regression is a standard technique based on maximum likelihood estimation (David & Stanley, 1989). The technique is based on the following equation,

(8) \pi(X)= \frac{e^{C_{0}+{C_{1}X}}}{1+e^{C_{0}+{C_{1}X}}}

where X is an independent variable which is any of the coupling metrics in our case and p is the probability of occurrence of a fault in a class, which is actually a dependent variable. We perform ULR, for each coupling metric, against the probability of occurrence of fault and determine if the measure is statistically related to a fault- proneness.

To assess the statistical significance of each independent variable in the model, the likelihood ratio *χ*^2^ test is used. Assuming the null hypothesis that the true coefficient of X is zero, the statistic follows a *χ*^2^ distribution with one degree of freedom. We test *p* = *P*(*χ*^2^ > *statistic*). If *p* is less than 0.05 then we consider X is significant.

A ULR is undertaken using all the six coupling metrics (i.e. CBO, Fan-in, Fan-out, RFC, Vovel-in, and Vovel-out) against the dichotomous dependent variable, i.e., *fp* and *nfp*. The ULR identified the significant independent variables. Table 6 shows the coefficient computed and the p-value for all six coupling metrics. It is clear from the table that all the six coupling metrics are significantly associated with fault proneness. The results are similar to the conclusion drawn by other studies (Subramanyam & Krishnan, 2003; Shatnawi, Li & Zhang, 2006; English et al., 2009; Kpodjedo et al., 2009). However, our experiment exclusively reports the effectiveness of the proposed vovel metrics.

**Table 6:** Overall results of the ULR using coupling metrics in the selected five datasets.

Datasets	Ce		CBO		Fan-in		Fan-out		RFC		Vovel-in		Vovel-out	
	Coeff.	*p*-Value	Coeff.	*p*-Value	Coeff.	*p*-Value	Coeff.	*p*-Value	Coeff.	*p*-Value	Coeff.	*p*-Value	Coeff.	*p*-Value
Eclipse JDT Core	0.051	0	0.066	0	0.028	0	0.357	0	0.16	0	0	0	0	0
Equinox framework	0.01	0	0.02	0	0.014	0.001	0.24	0	0.13	0.039	0	0.003	0	0
Apache Lucene	0.015	0	0.042	0	0.017	0	0.274	0	0.16	0	0.001	0	0	0
Mylyn	0.01	0	0.1	0	0.046	0	0.278	0	0.18	0	0.001	0	0	0
Eclipse PDE UI	0.12	0	0.101	0	0.087	0	−0.06	0.268	0.03	0.01	0.001	0	0	0.043

#### Correlation with vovel metrics

**Figure 10:**
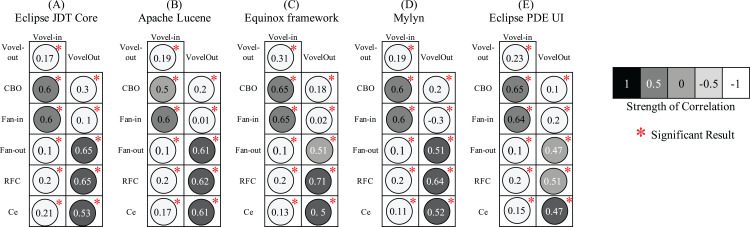
Spearman correlation coefficient of the coupling metrics with Vovel metrics.

The correlation analysis aims to determine empirically whether the proposed Vovel metrics are in consonance with the coupling metrics. The strong association implies the coverage of duplicate information. We perform Spearman correlation coefficient due to the nonparametric nature of the metrics, as we usually observe the skewed distribution of the design measures. The significance of the correlation was tested at a 95% confidence level. Figure 10 shows the correlation of the coupling metrics with Vovel metrics. As it can be seen that all of the associations are statistically significant and both Vovel metrics are not strongly correlated with any of the four coupling metrics. This implies the significant exclusive information coverage by the Vovel metrics. However, a mild correlation of *Vovel-in* with CBO and Fan-in is observed. Likewise, *Vovel-out* is slightly associated with Fan-out and RFC. The obvious reason is the consideration of the direction of method calls by the corresponding associating metrics.

#### Multivariate logistic regression

The MLR is usable where more than one metrics are to be analysed for their effect on predicting fault prone components. In this experiment we construct MLR for best fitting model to describe relationship between dependent and independent variable. The outcome of the MLR is fitted logistic regression equation.

(9) }{}log \frac{\pi (X)}{\pi X-1}=C_{0}+C_{1}X_{1}+C_{2}X_{2}+\cdot \cdot \cdot +C_{n}X_{n}

Since the objective of this experiment is to answer third research question, we construct following hypothesis.

**H0:** The proposed metrics do not improve the performance of SFP when used in combination of existing coupling metrics.

**H1:** The proposed metrics improve the performance of SFP when used in combination with existing coupling metrics.

We made two sets of features that act as independent variables. These sets and their corresponding elements are as follows:

*Set*_1_ = {*Ce*, *CBO*, *Fan − in, Fan − out, RFC}*

*Set*_2_ = *Set*_1_ ∪ {*Vovel − in, Vovel − out}*

We performed 10 experiments using the above set of independent variables (see Table 7).

**Table 7:** Descriptions of the experiments performed.

Sr. No.	Dataset	Independent variables	Dependent variables	Algorithm	Performance measure
1	Eclipse JDT Core	*Set*_1_	Binary (fp and nfp)	MLR	F1 Score, AUC, and MCC
2		*Set*_2_			
3	Equinox framework	*Set*_1_			
4		*Set*_2_			
5	Apache Lucene	*Set*_1_			
6		*Set*_2_			
7	Mylyn	*Set*_1_			
8		*Set*_2_			
9	Eclipse PDE UI	*Set*_1_			
10		*Set*_2_			

In all the cases, dependent variable is binary, which shows the *fp* or *nfp* classes. This is the most common dependent variable used in 70% of the SFP studies (Radjenović et al., 2013). We applied MLR to build model. Each time, we split the dataset for training and testing purposes. After that, we performed 10-folds cross-validation on the training set. Finally, the average model is run on the test set for computing F1 score, AUC, and MCC.

F1 score or F-measure utilizes the precision and recall of the test by computing their harmonic mean. Its value ranges from 0.0 to 1.0. It is relatively more robust (Rizwan, Nadeem & Sindhu, 2019) and skewness insensitive. AUC (Area under the receiver operating characteristic curve) represents the performance of a classification model at all classification thresholds. This curve plots two parameters, i.e., True Positive Rate and False Positive Rate. It ranges from 0 to 1. The Matthews correlation coefficient (MCC), produces a high score only if the prediction obtained good results in all of the four confusion matrix categories. Its value ranges from −1.0 to 1.0. In all three performance measures, a higher value is desirable. The results out of each set are shown in Table 8 in their corresponding column.

**Table 8:** Performance computed the five datasets using MLR.

Dataset	Set 1			Set 2			Coefficient	*p*-Values
	F1	AUC	MCC	F1	AUC	MCC		
Eclipse JDT Core	0.51	0.52	0.51	0.72	0.71	0.66	2.12	0.0002
Equinox framework	0.56	0.61	0.71	0.76	0.75	0.73	1.01	0.0012
Apache Lucene	0.73	0.57	0.72	0.89	0.67	0.89	1.12	0.0000
Mylyn	0.86	0.59	0.78	0.9	0.79	0.86	1.07	0.0003
Eclipse PDE UI	0.65	0.63	0.61	0.72	0.69	0.69	1.03	0.0001

Table 8 shows the rejection of null hypothesis (*H*_0_) in all the five selected datasets. This implies that proposed coupling metrics significantly improves the predictive performance. It is observed that by using Vovel metrics predictive performance improves in all five datasets.

## Threats to Validity

The results of our experiment allow us to associate Vovel metrics with SFP. Nevertheless, before we could accept the result, we would have to consider possible threats to its validity.

### Construct validity

We include the converge of content coupling in our proposed metrics, however, we could not parse it due to its difficult nature. If we would do so, the results will even be more promising. Hence, the impact of content coupling in SFP remains unrevealed.

### Internal validity

1. With regard to the size of the projects, sufficient comprehensible project size is taken. The projects of a very large size or very small size were ignored.
2. With regard to the measuring of metrics, we are dependent on Javaparser. Nevertheless, the correctness of the values is ensured by applying the same measurement technique to one of our own projects, it is necessary to evaluate the measurement procedure through some other measure.

### External validity

The selected open-source projects are developed in Java. The results may vary when using projects developed in languages other than Java.

## Conclusion and Future Work

In this study, we explored the effectiveness of coupling metrics in SFP. The literature depicts that coupling metrics are useful in SFP; more specifically, CBO, RFC, Fan-in, and Fan-out are the most used and useful coupling metrics. Moreover, we found that volume and levels of coupling are not covered by any of the existing coupling metric. Therefore, we proposed novel coupling metrics Vovel metrics, that incorporate the volume and levels of coupling. We investigated the unique information coverage by the proposed metrics using correlation coefficient, wherein the proposed metrics are found least correlated. This infers the unique information coverage by the proposed metric. This answers the first research question.

Later, we performed ULR and MLR. The outcome of ULR advocates the association of proposed metrics with software faults. Finally, we employed MLR to assess the exclusive effectiveness of proposed metrics at the class level. The results of F1 score, AUC, and MCC advocate the viable addition of the proposed metrics to the existing software metrics. The results of ULR and MLR infer the positive impact of volume and levels of coupling in SFP. This answers the second research question.

In this study, coupling due to volume has been considered, however, four other aspects of coupling stated by Yourdon & Constantine (1979), i.e., direct, local, obvious, and flexible coupling are yet to be evaluated by SFP community.

## Supplemental Information

10.7717/peerj-cs.590/supp-1 Supplemental Information 1 Eclipse JDT Core Dataset.

10.7717/peerj-cs.590/supp-2 Supplemental Information 2 Equinox Dataset.

10.7717/peerj-cs.590/supp-3 Supplemental Information 3 Lucence Dataset.

10.7717/peerj-cs.590/supp-4 Supplemental Information 4 Mylyn Dataset.

10.7717/peerj-cs.590/supp-5 Supplemental Information 5 Code to generate the scatterplot for the results.

10.7717/peerj-cs.590/supp-6 Supplemental Information 6 Code for the modeling of logistic regression and computing the *P*-value of the results.

10.7717/peerj-cs.590/supp-7 Supplemental Information 7 Code to generate the information about the statistical distribution in the dataset used in this study.

## Additional Information and Declarations

### Data Availability

The following information was supplied regarding data availability: Data and code are available in the Supplemental Files.
